# Clinical and Psychological Impact of COVID-19 on Maintenance Hemodialysis Patients: Hospitalization Burden, De Novo Anxiolytic Use, and Long-Term Survival

**DOI:** 10.3390/medicina62040744

**Published:** 2026-04-13

**Authors:** Ioana Adela Ratiu, Danut Dejeu, Ozana Hocopan, Corina Moisa, Gabriel Cristian Bako, Nicu Olariu, Mihaela Pal, Edy Hagi-Islai, Anamaria Ratiu, Mirela Indries, Elena Emilia Babeș, Cristian Adrian Ratiu

**Affiliations:** 1 Faculty of Medicine and Pharmacy, University of Oradea, 1st December Square 10, 410073 Oradea, Romania; ioana.ratiu@didactic.uoradea.ro (I.A.R.); ddejeu@uoradea.ro (D.D.); hocopan.ozana@rezident.uoradea.ro (O.H.); mirela.indries@didactic.uoradea.ro (M.I.); eebabes@uoradea.ro (E.E.B.); cristian.ratiu@didactic.uoradea.ro (C.A.R.); 2 Nephrology Department, Emergency Clinical Hospital Bihor County, 12 Corneliu Coposu Street, 410469 Oradea, Romania; drpalmihaela@yahoo.com; 3 Surgery Department, Emergency Clinical Hospital Bihor County, 12 Corneliu Coposu Street, 410469 Oradea, Romania; 4 Department of Pharmacy, Faculty of Medicine and Pharmacy, University of Oradea, 1st December Square 10, 410073 Oradea, Romania; 5 Department of Internal Medicine II, Division of Nephrology, “Victor Babeș” University of Medicine and Pharmacy, 300041 Timisoara, Romania; olariu.nicu@umft.ro; 6 Center for Molecular Research in Nephrology and Vascular Disease, Faculty of Medicine, “Victor Babeș” University of Medicine and Pharmacy, 300041 Timișoara, Romania; 7 Faculty of Dentistry, “Iuliu Hațieganu” University of Medicine and Pharmacy, Victor Babeş Street, No. 8, 400012 Cluj-Napoca, Romania; hagi.islai.edy@elearn.umfcluj.ro (E.H.-I.); ratiu.anamaria@elearning.umfcluj.ro (A.R.); 8 Infectious Diseases Department, Emergency Clinical Hospital Bihor County, 410087 Oradea, Romania; 9 Cardiology Department, Emergency Clinical Hospital Bihor County, 65 Gheorghe Doja Street, 410169 Oradea, Romania; 10 Discipline of Oral Implantology, Dentistry Department, Faculty of Medicine and Pharmacy, University of Oradea, 1st December Square 10, 410073 Oradea, Romania

**Keywords:** hemodialysis patients, COVID-19 pandemic, acute anxiety, anxiolytic treatment, mental health, hospitalization, COVID-19 vaccination

## Abstract

*Background and Objectives*: Hemodialysis (HD) patients represented a highly vulnerable population during the COVID-19 pandemic, both clinically and psychologically. Data regarding acute anxiety requiring pharmacologic treatment in this setting are limited. The aim of the study was to assess factors influencing clinical evolution, psycho-emotional disturbances reflected by “de novo” anxiolytic use, and vital prognosis of hospitalized COVID-19 patients on HD. *Materials and Methods*: The study included 211 patients followed between 2020 and 2023 (149 were COVID-19 positive and 80 required hospitalization) and comprised two sequential phases: an in-hospital phase during COVID-19, in which disease severity factors, in-hospital mortality, and the requirement for de novo anxiolytic therapy were assessed, and a follow-up phase, which evaluated overall mortality and the impact of vaccination on long-term outcomes. *Results.* Hospitalized patients were older, had lower dialysis adequacy, and a lower rate of COVID-19 vaccination. Severe COVID-19, associated with elevated inflammatory markers, prolonged hospitalization, and an increased need for anxiolytic therapy to control acute psychopathological disturbances, was significantly more frequent in patients with underlying oncological comorbidities. Patients who died from COVID-19 during hospitalization were older (69.364 ± 1.973 vs. 66.426 ± 1.546, *p* = 0.239), predominantly male (66.69% vs. 48.93%, *p* = 0.064), had similar BMI (26.836 ± 1.120 vs. 26.909 ± 0.943, *p* = 0.961), and had shorter duration on HD (5.182 ± 4.733 vs. 7.383 ± 6.060, *p* = 0.085). Patients who received anxiolytic therapy during hospitalization for COVID-19 were younger, predominantly male, and had a longer dialysis vintage as well as a higher body mass index. Although the de novo need for anxiolytics during COVID hospitalization was associated with multiple parameters in the linear regression analysis, the multivariable regression model showed a significant and strong association only with corticosteroid therapy (OR = 16.403, 95% CI = 4.433–62.111, *p* < 0.001). COVID-19 vaccination was associated with a significant reduction in mortality risk, with vaccinated patients exhibiting a 58% lower hazard of death compared with unvaccinated individuals (HR = 0.42; 95% CI: 0.28–0.62; *p* < 0.001). *Conclusions*: COVID-19 in HD patients is a multidimensional pathology, in which clinical severity and preventive strategies, such as vaccination, significantly influence survival. Acute anxiety requiring pharmacologic intervention was highly prevalent in hospitalized HD patients with COVID-19, but was not associated with worse survival (*p* = 0.903). Psychological burden should be recognized as an important component of care in this population.

## 1. Introduction

Hemodialysis (HD) dependence, as the terminal stage of chronic kidney disease (CKD), represents a significant public health challenge, both due to the continuously rising number of patients enrolled in HD programs and the high burden of associated comorbidities, which contribute to increased rates of hospitalization and mortality. The recent COVID-19 pandemic constituted a substantial stress test for this highly vulnerable population, profoundly affecting quality of life and leading to an exponential increase in mortality, either as a direct consequence of viral infection or indirectly through reduced access to healthcare services.

The frailty of patients with end-stage kidney disease (ESKD) results from a multitude of pathological conditions that are interrelated and act synergistically to the detriment of these patients. Severe cardiovascular alterations—including structural myocardial changes predisposing to fatal arrhythmias, myocardial infarction, and heart failure, as well as increased vascular stiffness and endothelial dysfunction—are the leading causes of morbidity and mortality in this population [1]. Chronic kidney disease–mineral and bone disorder (CKD–MBD) is a complex pathological condition with clinical consequences ranging from osteoarticular disease to exacerbation of cardiovascular pathology via vascular calcification [2,3]. Thus, a seemingly system-specific condition exerts widespread effects across the organism, further contributing to global physiological decline [4]. Endocrine–metabolic alterations, such as sarcopenic obesity and hypothyroidism, together with acute and chronic infections, present significant challenges in clinical diagnosis and management [5,6,7,8]. The immunological milieu of the dialysis patient is distinctive and characterized by a persistent inflammatory state [9,10]. In patients undergoing HD, the innate immune system is impaired, including dysfunction of monocytic and neutrophil cells, alterations in the complement system, abnormalities in cellular receptors, and cytokine excess [11,12]. In parallel, dysfunction of the adaptive immune system is evident, characterized by reduced B-lymphocyte counts, an increased Th1/Th2 ratio, and impaired antigen-presenting cell (APC) function [7].

Within this particular nosological context, dependence on HD exerts a substantial impact on the mental health of these patients [13]. Anxiety and depression are frequent psychiatric manifestations, while self-esteem and quality of life are severely impaired. Depression—characterized by a sustained loss of interest or pleasure for more than two weeks with negative social and familial consequences—is more common among HD patients. In the general population, the estimated prevalence of depression is approximately 5.7%, with higher rates observed in females and an increased prevalence in individuals over 70 years of age [14]. Anxiety, a mental disorder characterized by persistent and often unexplained fear in the absence of a clearly defined object or underlying cause, frequently overlaps with depression. However, unlike depression—whose prevalence and incidence in HD patients have been evaluated in longitudinal studies—anxiety has been less extensively investigated in this population. It is estimated that the prevalence of these two conditions is approximately 20% among patients undergoing HD, more than three times that observed in the general population [13].

Two fundamental challenges arise in the assessment and management of anxiety and depression in patients undergoing HD: the validation of diagnostic instruments specifically adapted to this population and the appropriate treatment of these conditions. The validated scales commonly used to diagnose depression and anxiety in the general population—such as the Beck Anxiety Inventory (BAI), Beck Depression Inventory (BDI), Hamilton Rating Scales, State–Trait Anxiety Inventory (STAI), Hospital Anxiety and Depression Scale (HADS), Patient Health Questionnaire (PHQ-9), and the 7-item Generalized Anxiety Disorder scale (GAD-7)—have not been specifically adapted to the clinical context of patients receiving HD. Consequently, reported prevalence and incidence rates of depression and anxiety are highly dependent on the assessment tools employed. Application of these instruments has revealed a prevalence of anxiety ranging from 22% to 55.38% and a prevalence of depression ranging from 29.4% to 50.77% [15,16,17]. The use of these assessment scales has demonstrated the major impact of anxiety and depression on hospitalization requirements and overall mortality among patients undergoing HD [16]. Factors contributing to deterioration in mental health status include demographic variables (such as duration of hemodialysis), nosological factors (such as coronary artery disease, heart failure, and type 2 diabetes mellitus), biological parameters (such as prealbumin and hemoglobin levels), and environmental factors [18]. For instance, elevated noise levels in hemodialysis units have been correlated with a higher incidence of anxiety and sleep disturbances [19].

A second critical challenge involves optimizing psychosocial and pharmacological treatment strategies for anxiety and depression in HD patients [20,21,22,23]. Current guidelines recommend selective serotonin reuptake inhibitors (SSRIs) and serotonin–norepinephrine reuptake inhibitors (SNRIs)—such as sertraline, escitalopram, fluoxetine, duloxetine, and venlafaxine—as first-line treatments, most of which undergo hepatic metabolism and have reduced renal clearance [24]. Clinical evidence indicates that the use of these agents is associated with a reduction in markers of chronic inflammatory status, such as hs-CRP, IL-1, and IL-6. In line with this approach, the European Renal Best Practice (ERBP) guidelines recommend administering selective SSRIs for an initial period of 8 to 12 weeks, followed by reevaluation. Additionally, dose adjustments are advised for agents such as selegiline, amitriptylinoxide, venlafaxine, desvenlafaxine, milnacipran, bupropion, reboxetine, and tianeptine in patients with CKD stages 3–5 [25,26]. Benzodiazepines represent a second-line treatment for anxiety, acting by enhancing gamma-aminobutyric acid (GABA) transmission, an endogenous anxiolytic. Consequently, they exert anxiolytic, hypnotic/sedative, muscle-relaxant, anticonvulsant, and anesthetic effects. They are primarily used in generalized anxiety disorders and specific phobias, being particularly useful in acute situations for short-term use due to the risk of dependence. Although their use is generally recommended to be limited to three months, long-term use has been reported in hemodialysis patients, with a prevalence of up to 14% [27,28]. Long-term benzodiazepine therapy in hemodialysis patients, beyond the inherent risk of dependence, has been associated with increased mortality, particularly among individuals diagnosed with chronic obstructive pulmonary disease [29]. Studies demonstrating the real-world efficacy of these therapies in hemodialysis patients are limited, and the correlation between treatment use and patient prognosis is also poorly defined.

The COVID-19 pandemic has led to a paradigm shift in the course of patients undergoing HD. The virus affected both young and elderly populations, increasing morbidity and mortality both directly and indirectly, through reduced access to specialized medical services. It also altered interpersonal relationships and led to particular psychosomatic changes, with a high prevalence of anxiety.

However, to date, few studies in the literature have specifically investigated changes in the psychological condition of HD patients during the pandemic, and even fewer have examined the use of anxiolytics in this context or the correlation between patients’ psychological status and their clinical prognosis and outcomes. Published intrapandemic studies have provided conflicting data. Bonenkamp et al. found no significant differences in anxiety, depression, or sleep quality among HD patients during the pandemic compared with the prepandemic period [30]. A meta-analysis of more than 68 studies from over 19 countries demonstrated an increased incidence of depression and anxiety among HD patients during the pandemic, with higher rates observed in younger patients, women, individuals from rural areas, and those with lower socioeconomic status [31]. Regarding the type of dialysis, Jin Young Yu (2021) identified notable differences in the incidence of anxiety, stress, and insomnia between HD and peritoneal dialysis patients during the pandemic, with higher rates observed in those undergoing HD [32].

In light of the large hemodialysis population under our care and our tertiary hospital’s role as a COVID-19 treatment center during the pandemic, the study aimed to evaluate the multidimensional impact of COVID-19 in a maintenance hemodialysis population, including hospitalization burden, acute psychological distress requiring pharmacologic treatment, and long-term survival, with particular focus on vaccination status.

## 2. Materials and Methods

We performed a retrospective observational single-center cohort study involving 211 patients enrolled in our hospital’s hemodialysis program during the pandemic. Patients were evaluated between January 2020 and December 2023. Data collected included COVID-19-related hospitalizations, clinical and laboratory parameters during hospitalization, administered medications, the need for in-hospital anxiolytics, prognosis, and mortality. The study was approved by the Ethics Committee SCJUBH (approval no. 2376/23.01.2025). Written informed consent for the use of personal data, with guaranteed anonymity, was obtained from all patients either upon hospitalization or during outpatient visits at our specialized hospital clinics.

### 2.1. Study Population

**Figure 1:**
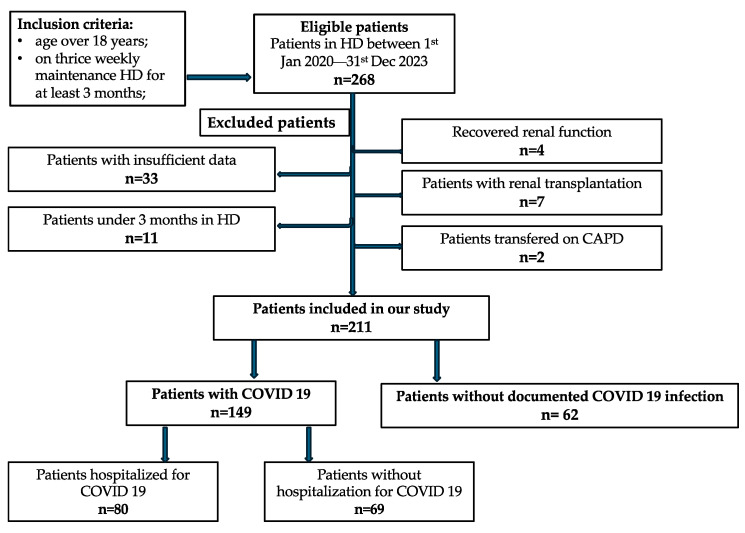
Flowchart of patient selection for study inclusion.

Among the 268 patients undergoing hemodialysis who were hospitalized in our unit between January 2020 and December 2023, 211 were eligible for enrollment in the study. Thirty-three patients were excluded due to missing data, 11 because they discontinued HD before three months, 4 due to partial recovery of renal function, 7 because they underwent kidney transplantation, and 2 because they transitioned to CAPD. Of these, 149 patients were diagnosed with active COVID-19 infection, and 80 of them required hospitalization. Anxiolytic therapy was initiated de novo during hospitalization in 52 patients (Figure 1).

Inclusion criteria: age > 18 years; receiving thrice-weekly maintenance hemodialysis for at least 3 months. Exclusion criteria: insufficient data, recovered renal function, renal transplantation, and transferred to CAPD.

### 2.2. Study Design

The study was divided into two sequential phases to assess both the acute and long-term impact of COVID-19 on this vulnerable population.

**Phase 1—In-hospital Outcomes**

Objective: To evaluate the clinical course and acute psychological impact during hospitalization.

Exposure of interest: De novo anxiolytic therapy initiated during hospitalization.

Outcomes: (a) Clinical severity markers; (b) prevalence of anxiolytic use; (c) in-hospital mortality (exploratory).

**Phase 2—Long-term Follow-up**

Objective: To assess long-term survival and the impact of COVID-19 vaccination.

**Figure 2:**
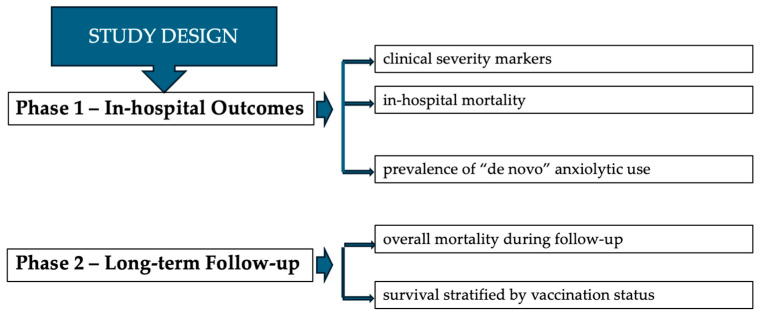
Flowchart of the study design.

Outcomes: (a) Overall mortality during follow-up; (b) survival stratified by vaccination status (Figure 2).

By structuring the study in two sequential phases, we were able to evaluate the acute clinical and psychological burden of COVID-19 during hospitalization and the long-term outcomes in the broader cohort, including the effect of vaccination. This approach enables a comprehensive assessment of both immediate and long-term effects of COVID-19 in maintenance HD patients.

Patients’ survival was evaluated using Kaplan–Meier curves and the log-rank test across subgroups defined by COVID-19 infection, hospitalization and vaccination status. Exploratory analyses examined correlations between clinical and demographic parameters and the primary or secondary outcomes to identify subgroups with potentially distinct clinical trajectories within this heterogeneous cohort.

Multivariable Cox regression was performed to assess the factors influencing survival in the entire cohort of 211 patients.

### 2.3. Definitions

COVID-19 infection was defined as a positive SARS-CoV-2 test. The diagnosis was initially established by rapid antigen testing and confirmed by detection of SARS-CoV-2 RNA in nasopharyngeal swab samples using real-time polymerase chain reaction (RT-PCR). The RT-PCR assay, performed on the NIMBUS_CFX96 analyzer, had a detection limit corresponding to a cycle threshold (Ct) of 40. Testing was conducted in symptomatic patients or those with known COVID-19 exposure. During the pandemic, PCR testing was routinely performed for hospitalized patients, while rapid antigen testing was used for symptomatic individuals or contacts of COVID-19-positive patients to enable prompt isolation and prevent viral spread.

**Severe COVID-19.** The definition of severe COVID-19, based on established criteria, included any of the following: respiratory distress with a respiratory rate ≥30 breaths per minute, or oxygen saturation ≤93% on room air whereas critically ill patients had a partial pressure of arterial oxygen to fraction of inspired oxygen ratio <300 mmHg, necessitate high-flow oxygen, mechanical ventilation and multi-organ disfunction co-related to COVID-19 infection [33].

### 2.4. Data Collection

Patients’ hospitalization records and outpatient evaluations were used in our study.

Data were collected from patients’ hospitalization records and outpatient evaluation charts. The collected parameters were as follows:

(i) Demographic and biometric data: age, gender, HD vintage, body mass index (BMI), body surface area (BSA).
(ii) Data regarding HD procedure: Kt/V, as a marker of HD efficacy, has a target between 1.2 and 1.4 in the case of patients receiving thrice-weekly HD. The HD machines available in our hospital were Fresenius 4008s and Braun Dialog+. During the study, we used synthetic dialyzers (Elisio/Nipro/Akita/Japan, FX-Fresenius/Saarland/Germany, Diacap Pro Braun/Hesse/Germany) with a surface area between 1.9 and 2.1 m^2^, ultrafiltration coefficient (Kuf) 75–82 mL/h/mmHg, and gamma ray sterilization.
(iii) Laboratory data: hemoglobin (Hb), albumin, total cholesterol, hormone (iPTH), calcium, phosphate, D dimer, procalcitonin. For Hb assessment, the UNICEL hematology analyzer DxH 900, Beckman Coulter, Danvers, MA, USA, was used, based on the impedance technique. Biochemical parameters such as hs-CRP, albumin, total cholesterol, calcium, phosphate, and iPTH were measured using a turbidimetric technique on the B04078-AU5811 chemistry analyzer, Beckman Coulter, Danvers, MA, USA. Procalcitonin was measured using the chemoluminescent microparticle immunoassay (CMIA) technique on the ALINITY AC03944 analyser (Abbott Laboratories, North Chicago, IL, USA).
(iv) Comorbidities: diabetes mellitus, arterial hypertension, coronary artery disease and malignancies.
(v) Medication during hospitalization: (a) antiviral medication included lopinavir/ritonavir (LPV/RTV) (Kaletra), favipiravir (FPV) (FluGuard), remdesivir (RDV) (Veklury), darunavir + ritonavir (Prezista + Norvir) (DRV/RTV), and molnupiravir (MPV) (Lagevrio); (b) the antibiotics represented by cefalosporins and carbapenems (c) anticoagulants: oral or low MW heparins. (d) corticotherapy: dexamethasone or HHC; (e) anxiolytics: lorazepam and clonazepam.

Statistical analyses were performed using Jamovi version 2.7.2.0 and verified with Stata/SE 17.0 for Windows (64-bit, Revision 21.05.2024), copyright 1985–2021 4905 Lakeway Drive, College Station, TX, USA (state licence no 401809208832). Continuous variables were first assessed for normality using the Shapiro–Wilk test and were compared using *t*-tests and Mann–Whitney U test, while categorical variables were analyzed with the Pearson Chi-square (χ^2^) test. Continuous data are presented as means ± standard deviations (SDs). Linear regression was applied to continuous outcomes, logistic regression to binary outcomes, and Cox proportional hazards regression for time-to-event outcomes. Model performance was evaluated using adjusted R^2^ and F-tests (linear), odds ratios with 95% CIs and AUC (logistic), and hazard ratios with 95% CIs and the concordance index (Cox). Variables included in the multivariable regression models were selected based on clinical relevance, evidence from the literature, and findings from univariate analyses. In addition to the primary multivariable logistic regression, the Generalized Linear Model (GLM) module in Jamovi was applied as a supplementary approach to confirm the robustness of the initial model. A binomial family with a logit link was specified, including the same predictor variables (age, hs-CRP, antiviral treatment, and COVID-19 vaccination). Coefficients were exponentiated to obtain odds ratios (OR) with 95% confidence intervals (CI), allowing direct comparison with the original logistic regression results. Additionally, Kaplan–Meier survival curves were generated to evaluate mortality over the study period, including during the COVID-19 pandemic. Multivariable Cox regression analysis was performed using IBM SPSS Statistics version 25 (IBM Corp., Armonk, NY, USA).

## 3. Results

### 3.1. Phase 1—In-Hospital Outcomes

#### 3.1.1. The Main Characteristics of Hospitalized Versus Non-Hospitalized COVID-19-Positive Patients Undergoing Chronic Hemodialysis: Overall Mortality

##### General Characteristics of COVID-19 HD Hospitalized Patients

Comparative assessment of hospitalized and non-hospitalized COVID-19-positive patients did not reveal significant differences in demographic characteristics, including age (67.638 ± 10.934 vs. 65.957 ± 12.756, *p* = 0.388), sex (57.5% vs. 49.27%, *p* = 0.766), or dialysis vintage (6.475 ± 5.625 vs. 7.662 ± 5.468, *p* = 0.197). No differences were observed regarding BMI (27.050 ± 6.412 vs. 26.043 ± 5.369, *p* = 0.394). The proportion of arteriovenous fistulas as the vascular access for hemodialysis was similar between hospitalized and non-hospitalized patients (67.5% vs. 68.11%, *p* = 0.936), and dialysis efficacy was comparable in both groups (Kt/V 1.524 ± 0.323 vs. 1.616 ± 0.293, *p* = 0.074). We observed similar proportions of patients with diabetes (36.25% vs. 30.43%, *p* = 0.453), hypertension (88.75% vs. 94.2%, *p* = 0.240), and ischemic heart disease (42.5% vs. 42.02%, *p* = 0.954) among hospitalized and non-hospitalized COVID-19-positive patients.

We do not find statistically significant differences between hospitalized and non-hospitalized patients regarding hemoglobin level (10.636 ± 1.616 vs. 10.713 ± 1.397, *p* = 0.758) or other laboratory parameters, including albumin (3.68 ± 0.605 vs. 3.811 ± 0.388, *p* = 0.129), calcium (8.88 ± 0.988 vs. 8.968 ± 0.94, *p* = 0.585), phosphorus (5.193 ± 2.058 vs. 5.037 ± 1.626, *p* = 0.62), and iPTH (321.15 ± 326.56 vs. 387.75 ± 357.67, *p* = 0.254). The only laboratory marker that was significantly elevated in hospitalized patients was CRP (109.121 ± 99.298 vs. 23.568 ± 31.89, *p* < 0.001). Antiviral therapy was administered to 92.5% of hospitalized patients (*p* < 0.001), whereas vaccination was significantly more common among non-hospitalized COVID-19 HD patients (*p* < 0.001) (Table 1).

The antiviral agents administered during hospitalization, depending on their availability at the time of admission, included lopinavir/ritonavir (LPV/RTV) (Kaletra), favipiravir (FPV) (FluGuard), remdesivir (RDV) (Veklury), darunavir + ritonavir (Prezista + Norvir) (DRV/RTV), and molnupiravir (MPV) (Lagevrio). The evaluation of patients who received each specific antiviral agent, compared with all other patients, did not reveal significant differences in age, BMI, or length of hospitalization (Table S1).

##### Characteristics of Hospitalized HD Patients with Severe COVID-19

Although not statistically significant, patients with severe COVID-19 tended to be younger (66.861 ± 1.882 vs. 68.273 ± 1.616, *p* = 0.569), predominantly male (66.66% vs. 54.54%, *p* = 0.555), and had a higher BMI (27.886 ± 1.137 vs. 26.055 ± 0.906, *p* = 0.206). Dialysis duration was similar between groups (6.028 ± 0.914 vs. 6.841 ± 0.871, *p* = 0.534). At the threshold of statistical significance, diabetes (*p* = 0.065) and ischemic heart disease (*p* = 0.093) were more frequently observed among patients who developed severe COVID-19. Severe disease occurred significantly more often in patients with malignancies undergoing hemodialysis (*p* = 0.014). Hemoglobin (10.357 ± 0.267 vs. 10.86 ± 0.243, *p* = 0.164) and albumin levels (3.581 ± 0.125 vs. 3.761 ± 0.068, *p* = 0.189) were lower in patients with severe COVID-19, but the differences did not reach statistical significance. Among specific inflammatory markers, C-reactive protein (144.066 ± 16.799 vs. 80.408 ± 13.479, *p* = 0.004) and procalcitonin (8.499 ± 2.787 vs. 2.444 ± 0.669, *p* = 0.031) were significantly elevated in severe cases, whereas D-dimer levels, despite being higher in severe forms, did not reach statistical significance (1908.68 ± 1788.47 vs. 1702.074 ± 1530.948, *p* = 0.676).

Antiviral therapy, which is most effective in the early days of COVID-19 infection, was administered more frequently to patients with moderate COVID-19 (86.11% vs. 97.72%, *p* = 0.05). Carbapenems were the antibiotics of choice in severe cases (66.66% vs. 31.81%, *p* = 0.002), whereas cephalosporins predominated in patients with moderate in-hospital COVID-19 (30.55% vs. 54.54%, *p* = 0.031). Anticytokine therapy was administered to 16% of patients with severe COVID-19 (*p* = 0.072), and anticoagulant therapy was provided in most cases, in the absence of contraindications. Only a small proportion of patients with severe disease had been vaccinated against COVID-19 (16.66% vs. 25%, *p* = 0.365). The duration of hospitalization was significantly extended in severe forms of COVID-19 (14.75 ± 6.909 vs. 10.477 ± 6.54, *p* = 0.006), as was the mortality associated with SARS-CoV-2 infection (63.88% vs. 27.72%, *p* = 0.001) (Table 2).

##### Comparative Evaluation of Mortality in Hospitalized COVID-19 HD Patients

Patients who died from COVID-19 during hospitalization were older (69.364 ± 1.973 vs. 66.426 ± 1.546, *p* = 0.239) and predominantly male (66.69% vs. 48.93%, *p* = 0.064), with similar BMI (26.836 ± 1.120 vs. 26.909 ± 0.943, *p* = 0.961) and shorter duration on HD (5.182 ± 4.733 vs. 7.383 ± 6.060, *p* = 0.085). There were no significant diffrences regarding vascular access (66.66% vs. 68.08%, p0.894), HD efficiency (Kt/V = 1.501 ± 0.357 vs. 1.540 ± 0.299, *p* = 0.596), or the presence of comorbidities, including diabetes (39.39% vs. 34.04%, *p* = 0.624), coronary artery disease (48.48% vs. 38.29%, *p* = 0.364), or malignancies (24.24% vs. 14.89%, *p* = 0.292). Laboratory analyses revealed decreased levels of hemoglobin (10.410 ± 1.562 vs. 10.794 ± 1.651, *p* = 0.299), calcium (8.624 ± 1.007 vs. 9.060 ± 0.943, *p* = 0.051), and albumin (3.529 ± 0.757 vs. 3.786 ± 0.450, *p* = 0.061), alongside significantly higher levels of C-reactive protein (151.515 ± 116.068 vs. 79.356 ± 73.262, *p* = 0.001) and procalcitonin (10.425 ± 3.507 vs. 2.382 ± 0.591, *p* = 0.005). No differences were noted in the distribution of antiviral treatments, except for remdesivir, which was used more frequently among patients with unfavorable outcomes (24.24% vs. 6.38%, *p* = 0.022). The percentage of deceased patients vaccinated against SARS-CoV-2 was significantly lower than that among patients with favorable clinical outcomes (6.06% vs. 31.91%, *p* = 0.005) (Table 3).

The multivariable regression model for mortality predictors was based on variables identified from univariate analyses and clinically relevant confounders. Among 81 patients with 33 deaths, several four-predictor models were tested for stability.

Laboratory parameters differing between groups included calcium, CRP, albumin, and procalcitonin, with negligible collinearity between CRP and procalcitonin (VIF = 1). In the multivariable model, CRP was a significant predictor of mortality (OR = 1.015, 95% CI 1.006–1.024, *p* < 0.01), whereas procalcitonin was not (OR = 1.015, 95% CI 0.983–1.211, *p* = 0.101). Calcium and albumin also did not reach statistical significance (*p* = 0.094 and 0.117; 95% CIs included 1), despite suggesting a potential >50% reduction in mortality risk (OR = 0.649 and 0.524). These results underscore CRP as the most reliable laboratory marker of disease severity and mortality in this cohort.

Clinically relevant variables included age, diabetes, duration of hemodialysis, malignancy, length of hospital stay, vaccination, and antiviral treatment, with the last three showing statistical significance in univariate analysis. To identify clinically relevant predictors, a multivariable regression model for COVID-19 mortality was constructed including all candidate parameters. The model suggested a potential impact of age, sex, and malignancy on COVID-19 death, although none of these variables reached statistical significance: age (OR = 1.029, 95% CI 0.983–1.078, *p* = 0.222), sex (OR = 2.296, 95% CI 0.855–6.167, *p* = 0.099), and malignancy (OR = 2.201, 95% CI 0.625–7.752, *p* = 0.219).

Subsequently, three regression models were constructed, in which these parameters were introduced sequentially, alongside CRP as a marker of disease severity, vaccination status, and antiviral treatment—all variables shown to be significant in univariate analysis.

In the first model, age, CRP, vaccination status, and antiviral treatment were included. The model demonstrated a total explained variance of 23.1% (McFadden’s R^2^ = 0.231), an overall fit statistic (AIT) of 93.369, and an AUC of 0.795. The analysis identified a major impact of CRP (OR = 1.008, 95% CI 1.002–1.014, *p* = 0.008), antiviral treatment (OR = 0.058, 95% CI 0.004–0.813, *p* = 0.035), and COVID-19 vaccination (OR = 0.179, 95% CI 0.032–0.899, *p* = 0.037) on mortality (Table 4).

The second model included sex, CRP, vaccination status, and antiviral treatment. This model showed an AIC of 93.933, R^2^ = 0.226, and an AUC of 0.802. In this alternative model, replacing age with sex, the antiviral therapy lost statistical significance, so that only CRP (OR = 1.008, 95% CI 1.002–1.014, *p* = 0.009) and COVID-19 vaccination (OR = 0.155, 95% CI 0.028–0.864, *p* = 0.033) remained significant.

A third model was constructed, which included malignancy alongside CRP, vaccination status, and antiviral treatment. This model demonstrated an AIC of 95.895, R^2^ = 0.208, and an AUC of 0.778. It also confirmed the impact of CRP (OR = 1.007, 95% CI 1.002–1.013, *p* = 0.008), COVID-19 vaccination (OR = 0.169, 95% CI 0.031–0.925, *p* = 0.040), and antiviral treatment (OR = 0.080, 95% CI 0.007–0.923, *p* = 0.043) on mortality. However, given the limited number of cases and the low prevalence of malignancy in the cohort, we opted to present the model including age, CRP, COVID-19 vaccination, and antiviral treatment. Age was selected as a predictor because it is the most commonly used clinical parameter in statistical models of COVID-19 mortality, and its inclusion helps ensure model stability and interpretability in the context of a relatively small sample size.

Furthermore, applying the GLM module for multivariable regression did not materially alter the results of the previous model, with all key parameters retaining their statistical significance and overall relevance, while age approached borderline significance. Although the magnitude of the associations was attenuated (CRP: OR 1.008 to 1.002; antiviral treatment: OR 0.058 to 0.607; vaccination: OR 0.179 to 0.749), the direction of the effects remained unchanged, supporting the robustness and consistency of the findings (Table 5).

**Figure 3:**
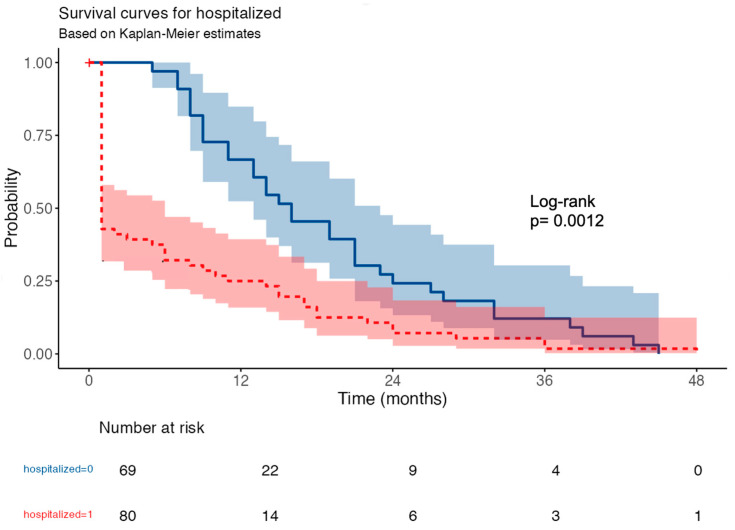
Kaplan–Meier survival analysis among hospitalized and nonhospitalized COVID-19 HD patients.

Kaplan–Meier survival analysis revealed significantly higher mortality in hospitalized COVID-19 patients compared to non-hospitalized HD patients (log-rank *p* = 0.0012) (Figure 3).

Univariable Cox proportional hazards analysis showed that hospitalization was significantly associated with an increased risk of death (HR = 2.18; 95% CI: 1.41–3.38; *p* < 0.001).

#### 3.1.2. Anxiolytic Treatment During COVID-19 Hospitalization in HD Patients

##### Characteristics of HD Patients Who Require De Novo Anxiolytics During Hospitalization for COVID-19

The anxiolytic agents used during hospitalization included alprazolam and clonazepam, and, in extreme situations, haloperidol. Patients who received anxiolytic therapy during hospitalization for COVID-19 were younger, predominantly male, and had a longer dialysis vintage as well as a higher body mass index. Hemoglobin levels, serum albumin, Kt/V, or C-reactive protein did not differ significantly between groups. Corticosteroid therapy had a significant impact on anxiolytic requirements. Notably, anxiolytic use was significantly more prevalent among patients with severe COVID-19 and those admitted to intensive care units; however, no statistically significant differences were observed in COVID-19-related mortality (Table 6).

##### Predictors for Anxiolytic Requirement in HD Patients During Hospitalization for COVID-19

The multivariable regression model was primarily based on findings from univariate analyses. Among the four variables significantly associated with the need for anxiolytics—length of hospital stay, COVID-19 severity, ICU admission, and corticosteroid therapy—corticosteroid therapy and severe COVID-19 were selected, as the other variables were encompassed by the latter. Additionally, BMI, which is clinically relevant and demonstrated borderline significance in the univariate analyses, hemoglobin level, given its potential impact on developing depression, and gender, due to the higher prevalence of depression among females, were also included in the model.

The number of covariates was limited to five, appropriate given the sample size (52 out of 81 patients receiving anxiolytics) to avoid overfitting. The model with the best characteristics had 0.825 accuracy, 0.643 specificity, 0.923 sensitivity, AIC 83.859, AUC 0.834, and R^2^McF 0.306. Model selection was guided by statistical criteria, including reductions in deviance and comparisons of Akaike Information Criterion (AIC) values, and the model with the lowest AIC was chosen to balance fit and complexity. This approach ensured a parsimonious and clinically meaningful model.

We also explored alternative models replacing Kt/V and BMI with age and gender. These models showed a slightly poorer fit, but the association between corticosteroid therapy and anxiolytic use remained similar. The final model has an AIC of 79.993 and R^2^McF of 0.306. Variance Inflation Factor (VIF) values for all covariates ranged from 1.014 to 1.062, indicating no significant multicollinearity and supporting the stability of the model estimates. Additionally, the final multivariable logistic regression model demonstrated good explanatory performance, with a Nagelkerke R^2^ of 0.451, suggesting that approximately 45% of the variability in anxiolytic requirement was explained by the included predictors.

**Figure 4:**
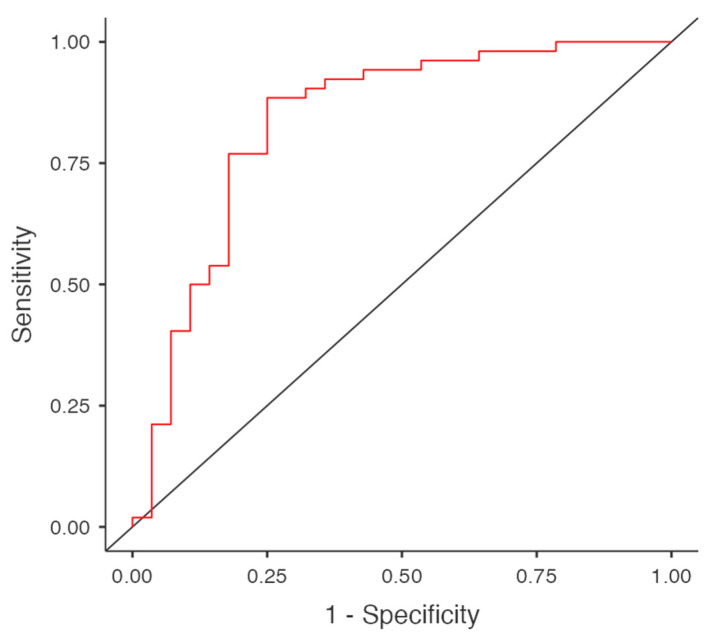
The multivariable regression model ROC curve for anxiolytic treatment during hospitalization.

The ROC curve is represented below (Figure 4).

Corticosteroid therapy was strongly associated with the use of anxiolytic therapy during hospitalization, conferring a nearly 17-fold increase in odds (OR 16.403, 95% CI 4.332–62.111, *p* < 0.001) (Table 7).

To further evaluate the robustness of the model, a generalized linear model (GLM) was applied as a sensitivity analysis. Using the GLM module for multivariable regression, corticosteroid therapy was the only variable significantly associated with the outcome, with treated patients having 80% higher odds (OR = 1.805, 95% CI 1.464–2.228, *p* < 0.001). In contrast, BMI (OR = 1.007, 95% CI 0.993–1.022, *p* = 0.357), hemoglobin (OR = 0.999, 95% CI 0.945–1.055, *p* = 0.974), severe COVID-19 form (OR = 1.096, 95% CI 0.909–1.322, *p* = 0.329), and gender (OR = 1.031, 95% CI 0.864–1.230, *p* = 0.733) were not significantly associated with the outcome. These results are consistent with findings from classical multivariable logistic regression, confirming that corticosteroid therapy is the main determinant of the outcome, while other clinical and demographic variables do not contribute independently. The concordance across modeling approaches supports the robustness and internal validity of the results (Table 8).

Given the high OR in the anadjusted multivariable model and the wide 95% CI, we then attempted to assess how the selected parameters influenced the OR value and the 95% CI. We designed two separate models. In the first model, we included only corticosteroid therapy. The OR was 21.6 and the 95%CI = 6.007–77.674. Subsequently, we adjusted for confounders. Adjustment for disease severity resulted in a 17.6% attenuation of the association between corticosteroid therapy and anxiolytic requirement (the OR decreased from 21.6 to 17.79), suggesting partial confounding but persistence of a strong independent association.

In addition, to clarify the large OR of 17.392 and the wide confidence interval, we present the distribution of patients according to corticosteroid and anxiolytic use in a **2 × 2 contingency table** (Table 9). The table shows that the number of patients receiving anxiolytics without corticosteroids (n = 4) and those receiving corticosteroids without anxiolytics (n = 10) is small. This indicates that events are relatively rare in these groups, which can result in large OR estimates and wide confidence intervals.

Therefore, OR = 16.403 reflects a strong association between corticosteroid use and the need for anxiolytics; however, the precision of this estimate is limited by the sample size and the low frequencies in certain cells. This limitation should be considered when interpreting the results, and future studies with larger samples are needed to confirm this association.

### 3.2. Phase 2—Long-Term Follow-Up

#### 3.2.1. Comparative Characteristics of COVID-Positive and COVID-Negative Patients in a Hemodialysis Program: Overall Mortality During the Pandemic in HD Patients

COVID-positive patients had a significantly higher mean age compared with those who were not diagnosed with COVID-19, 66.859 ± 11.802 vs. 62.774 ± 2.044, *p* = 0.042. In addition, COVID-19 infection was associated with a significantly higher body mass index (BMI): 26.492 ± 5.947 vs. 24.156 ± 6.501, *p* = 0.012. On the other hand, no statistically significant differences were observed regarding sex distribution (56.37% vs. 54.83%, *p* = 0.838), duration of hemodialysis (7.020 ± 5.567 vs. 6.694 ± 5.312, *p* = 0.695), vascular access (67.78% vs. 61.29%, *p* = 0.695), or hemodialysis adequacy (1.567 ± 0.312 vs. 1.591 ± 0.323, *p* = 0.662). There were no differences in the incidence of diabetes mellitus (33.55% vs. 29.03%, *p* = 0.522), arterial hypertension (91.27% vs. 90.32%, *p* = 0.826), ischemic coronary artery disease (42,28% vs. 37.09%, *p* = 0.485), or malignancies (16.77% vs. 14.51%, *p* = 0.684).

From a paraclinical perspective, no differences were observed in serum hemoglobin levels (10.671 ± 1.514 vs. 10.778 ± 1.472, *p* = 0.641), calcium (8.920 ± 0.964 vs. 8.880 ± 0.922, *p* = 0.782), phosphorus (5.123 ± 1.872 vs. 4.995 ± 1.804, *p* = 0.653), albumin (3.740 ± 0.520 vs. 3.723 ± 0.371, *p* = 0.817) or intact parathyroid hormone (PTH) levels (329.693 ± 320.6 vs. 432.2 ± 577.151, *p* = 0.212). The proportion of vaccinated patients was similar between COVID-positive and COVID-negative patients (34.89% vs. 46.77%, *p* = 0.106) (Table 10).

**Figure 5:**
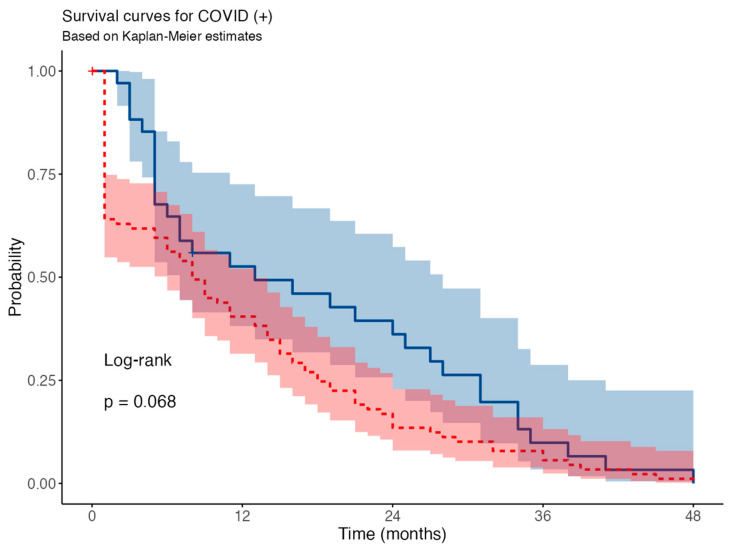
The Kaplan–Meier curve for the assessment of survival in HD COVID-19-positive versus negative patients.

During follow-up, Kaplan–Meier survival curves suggested lower survival in COVID-19-positive patients versus COVID-19-negative patients, though the difference was not statistically significant (log-rank *p* = 0.068) (Figure 5).

In univariable Cox proportional hazards analysis, COVID-19 positivity was associated with an increased risk of the event, with a hazard ratio (HR) of 1.49 (95% confidence interval [CI]: 0.99–2.24). This association was borderline statistically significant (*p* = 0.053), suggesting a trend toward worse outcomes in COVID-19-positive patients.

For the Cox multivariable regression model, the variables included in the model were selected based on their clinical relevance, evidence from the literature, and correlation tests: age, BMI, COVID-19 status, nutritional status (albumin), hemoglobin level, dialysis-related parameters (Kt/V and HD vintage), which may influence patient prognosis. Additionally, COVID-19-related variables (severe disease, vaccination status, and antiviral treatment) were incorporated in order to assess their independent impact on the risk of the outcome. The omnibus test of model coefficients showed that the model with the predictors was statistically significant compared to the null model (χ^2^(9) = 27.83, *p* = 0.001), indicating an overall improvement in model fit. The change in −2 log-likelihood was also significant (Δχ^2^ = 26.60, df = 9, *p* = 0.002), with a final −2 log-likelihood of 598.65.

In the Cox regression model, severe disease was independently associated with increased risk (HR = 2.086, *p* = 0.011, 95%CI 1.180–3.686), while vaccination had a protective effect (HR = 0.472, *p* = 0.009, 95%CI 0.269–0.826). No other variables reached statistical significance (Table 11).

#### 3.2.2. Vaccination Efficacy

**Figure 6:**
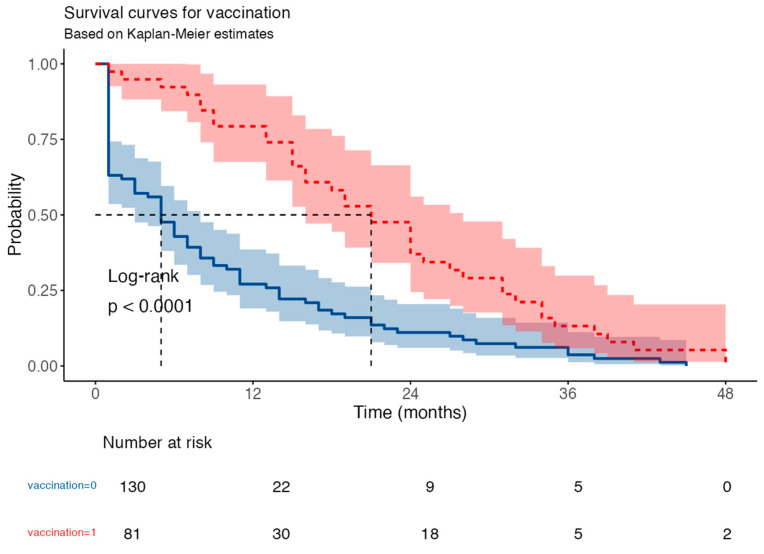
The Kaplan–Meier curve for the assessment of survival in HD COVID-19-vaccinated versus unvaccinated patients.

The Kaplan–Meier curve for the assessment of survival in COVID-19-vaccinated versus unvaccinated patients demonstrates the clear effectiveness of vaccination in preventing COVID-19-related mortality (log-rank *p* < 0.001) (Figure 6). Univariable Cox proportional hazards analysis indicated that COVID-19 vaccination was associated with a significant reduction in mortality risk, with vaccinated patients exhibiting a 58% lower hazard of death compared with unvaccinated individuals (HR = 0.42; 95% CI: 0.28–0.62; *p* < 0.001).

Survival differences may partly be due to the predominance of milder SARS-CoV-2 variants in later pandemic waves, which coincided with higher vaccination coverage.

## 4. Discussion

Compared with the general population, COVID-19 infection had a particularly profound impact on patients undergoing HD, representing a true turning point in their clinical course and resulting in unprecedented rates of morbidity and mortality. In this study, we highlighted the detrimental effects of SARS-CoV-2 on HD patients by analyzing clinical, laboratory, and outcome differences among hospitalized and non-hospitalized patients, those with severe COVID-19, and those requiring anxiolytic therapy. Our findings confirm the heightened vulnerability of this population, demonstrating significant rates of hospitalization and mortality that were associated with disease severity, inflammatory status, comorbidities, and the need for anxiolytic treatment. Furthermore, the data underscore the protective effect of COVID-19 vaccination on survival among these patients.

### 4.1. Hospitalization of HD Patients in the Context of SARS-CoV-2 Infection

Particularly during the initial phase of the pandemic, when the virulence of SARS-CoV-2 posed a major challenge for both patients and healthcare systems, the majority of HD patients diagnosed with COVID-19 required hospitalization. This was driven not only by the severity of respiratory involvement, including respiratory failure and the need for oxygen therapy, but also by efforts to limit viral transmission within an already highly vulnerable population. In our cohort, comparison between hospitalized and non-hospitalized patients did not reveal significant demographic differences. However, hospitalized patients were, on average, older, more frequently male, had higher BMI, and a slightly higher prevalence of diabetes mellitus, similar to other studies [34]. From a paraclinical perspective, hospitalized patients exhibited signs of systemic inflammation, as reflected by significantly elevated CRP levels, and protein-energy malnutrition, as evidenced by hypoalbuminemia, while no significant differences were observed in anemia severity or in phosphate–calcium balance parameters. Furthermore, hospitalized patients demonstrated lower Kt/V values, a lower prevalence of arteriovenous fistulas and vascular access, and markedly lower COVID-19 vaccination rates. Despite antiviral therapy, most studies indicate that mortality among hospitalized patients was high, both during hospitalization and in the long term. was approximately twice that of non-hospitalized patients [35,36,37]. In our patient cohort, mortality among hospitalized COVID-19 patients during the study period was approximately twice that of non-hospitalized patients. The impact of inpatient isolation, the uncertainty associated with the highly unpredictable course of the disease, and the overall frailty of this patient population contributed to the frequent use of anxiolytic therapy as part of the therapeutic arsenal in the majority of hospitalized hemodialysis patients.

### 4.2. Severe Forms of COVID-19 in HD Patients

HD patients present unique demographic, nosological, and immunological characteristics that render them particularly vulnerable to severe infections. These patients are typically older, reflecting both an aging HD population and the longer survival in recent decades. Advanced age is associated with immunosenescence, compounded by the immunodeficient status characteristic of end-stage kidney disease (ESKD) patients. Chronic inflammation is evident in this population, as indicated by elevations in common inflammatory markers and in more sensitive indicators, including proinflammatory cytokines, cystatin C, and leptin [12,38]. Additionally, a substantial proportion of HD patients have diabetes, which contributes to multiple metabolic disturbances. Cardiovascular pathology, whether associated with or independent of these factors, represents a major cause of mortality. Endothelial inflammation, structural and functional cardiac alterations, electrolyte imbalances predisposing to arrhythmias, and a high prevalence of heart failure all contribute to the heightened risk of death in this population [39]. Chronic viral infections, particularly hepatitis B and C, remain highly prevalent among hemodialysis patients in endemic regions, thereby contributing to amplification of the chronic proinflammatory state [40].

In the subgroup of patients who developed severe COVID-19 during hospitalization, the majority were male, with ages comparable to or even slightly lower than those with moderate forms of the disease. However, these patients had higher BMI and a markedly higher prevalence of diabetes, coronary artery disease, and oncological conditions. Despite a slightly shorter duration on HD compared to patients with moderate disease, laboratory profiles were largely similar with respect to anemia, phosphate–calcium balance, and albumin levels. Notably, inflammatory biomarkers, including CRP and procalcitonin, were significantly elevated in patients with severe disease.

Antiviral therapy was administered in accordance with national health programs. Early in the pandemic, remdesivir was reserved for patients with extremely severe disease; in our cohort, it was administered to approximately 1 in 5 patients with severe COVID-19. The majority of patients received lopinavir/ritonavir (LPV/RTV; Kaletra). Molnupiravir, which became available later in our country’s therapeutic arsenal, was predominantly administered to patients developing primarily moderate or mild disease toward the later stages of the pandemic.

To address all aspects of the inflammatory cascade, treatment regimens for COVID-19-infected patients included, in addition to etiologic antiviral therapy, anticoagulants, corticosteroids, and antibiotics to manage secondary bacterial infections. Anticoagulant therapy was used both for prophylaxis and treatment of COVID-19-associated thrombotic events, as well as for its anti-inflammatory, anti-complement, and endothelial-protective effects. Anticoagulant therapy plays a pivotal role in COVID-19 management by mitigating the prothrombotic state induced by SARS-CoV-2, thereby reducing thromboembolic complications and potentially improving survival, particularly in high-risk populations. In addition, it exerts anti-inflammatory, anti-complement, and endothelial-protective effects. In our cohort, low-molecular-weight heparins (LMWHs) were administered subcutaneously in the vast majority of patients with severe or moderate COVID-19, in the absence of contraindications. LMWH is known to possess superior anti-inflammatory properties compared with other anticoagulants. Patients who had previously received vitamin K antagonists or non-vitamin K oral anticoagulants (NOACs) continued their prior therapy in the absence of COVID-related adverse effects.

Anti-cytokine therapy used monoclonal antibodies targeting key proinflammatory interleukins, specifically anti-IL-6 (tocilizumab) and anti-IL-1 (anakinra). Tocilizumab is a humanized monoclonal antibody directed against the interleukin-6 receptor, administered intravenously to hospitalized patients with severe COVID-19 to mitigate the hyperinflammatory response associated with disease progression. Clinical studies have shown that early administration, within the first days, is effective in critical cases [41,42]. Anakinra (Kineret^®^) is a recombinant interleukin-1 receptor antagonist administered subcutaneously that blocks both IL-1α and IL-1β, cytokines that play a central role in systemic inflammation. The use of these therapies was limited by restricted availability and the high associated costs. Their efficacy has been assessed in several clinical trials, but the findings remain inconclusive [43,44]. In hospitalized patients, antibiotic therapy was primarily based on cephalosporins. In patients with severe COVID-19, broader-spectrum antibiotics were required, with carbapenems administered to nearly two-thirds of these patients.

### 4.3. Changes in Psychoemotional Behavior in the Context of COVID-19

In HD patients, the relationship between anxiety and COVID-19 infection is bidirectional: COVID-19 prevalence was higher among anxious patients, and those infected subsequently developed varying degrees of anxiety and depression [45]. Multiple factors contributed to the emergence of these psychoemotional changes. First, patients faced exposure to a novel, highly aggressive virus with an unpredictable disease course, affecting a population already extremely vulnerable. The high mortality rates observed in the initial phase of the pandemic were accompanied by a significant psychological burden at the time of diagnosis. Fear of transmitting the virus to family members further contributed to psychological stress, with studies showing that the degree of depression was higher among married individuals compared to those who were unmarried. Additionally, the lack of targeted treatment, and in the early stages of the pandemic, the absence of vaccination, represented key elements in the pathogenic chain leading to psychological disturbances in this population.

Anxiety was also attributable to hospitalization itself, particularly in the context of a disease requiring strict monitoring and isolation measures [46]. In this context, access to social support was restricted to healthcare personnel, who were equipped with protective gear that depersonalized them, and the time they could devote to each patient was minimized to the utmost extent possible [47]. Prolonged periods of isolation also negatively affect well-being [48]. Assessing mental health in this population is therefore essential, given the substantial effects of anxiety and depression on family and social functioning, treatment adherence, quality of life, and overall survival.

On the other hand, immune system alterations induced by SARS-CoV-2 infection may correlate with changes in psychological patterns. Moreover, COVID-19 treatment—which in the vast majority of cases included corticosteroid therapy, with its well-known psychiatric side effects such as corticosteroid-induced psychosis and insomnia—may contribute to the development of anxiety. The mechanisms by which corticosteroid therapy contributes to the development of psychological disorders remain insufficiently understood. In response to stress, the body activates an immediate reaction characterized by catecholamine release and the “fight-or-flight” response, followed by a slower response mediated through the limbic–hypothalamic–pituitary–adrenal (HPA) axis. This neuroendocrine activation facilitates the consolidation of fear as an adaptive defensive mechanism [49].

Psychiatric manifestations associated with corticosteroid use range from mild mood disturbances to severe conditions such as depression, anxiety, mania, and psychosis. These symptoms typically emerge within 3–4 days of initiating therapy and generally resolve within several weeks of discontinuation. Evidence suggests that not only treatment duration but also dosage—particularly doses exceeding 40 mg/day—is correlated with an increased risk of psychiatric adverse effects. Moreover, studies indicate that psychological symptoms, especially anxiety, may persist even after cessation of corticosteroid therapy [50].

Furthermore, it has been demonstrated that anxiety contributes to disease severity by exacerbating hypoxemia. Emotional stress, in turn, alters neuroendocrine system activity, with increased levels of IL-1ß, IL-2R, IL-6, IL-17, and TNF-α observed in patients with chronic depression [51]. This alteration in the immune pattern induced by SARS-CoV-2 overlays the immunological profile characteristic of patients with ESKD, which is marked by a proinflammatory state. Anxiety also induces hyperactivity of the autonomic nervous system, thereby increasing cardiac workload and the risk of acute pulmonary edema in hemodialysis patients, in whom this occurs on a morphofunctionally altered cardiovascular system further complicated by interdialytic volume overload, heparin use, electrolyte imbalances, and protein malnutrition. In this context, the role of depression and anxiety as negative prognostic markers in COVID-19 progression, versus their role as aggravating factors in disease evolution, remains to be established. Finally, it has been demonstrated that the onset of acute psychological stress in the setting of severe illness can evolve into a chronic condition, including PTSD, underscoring the critical importance of early diagnosis and appropriate management of anxiety in similar contexts [52].

In our patient cohort, in the absence of clear statistics and a definitive diagnosis recorded on discharge summaries, data regarding psychological status were inferred from the need for anxiolytic treatment. Thus, two-thirds of hemodialysis patients hospitalized for SARS-CoV-2 infection, and over four-fifths of patients with severe COVID-19—most of whom were transferred to ICU wards—required anxiolytic therapy. Administration of these medications was carried out with caution due to the risk of oversedation, potential drug interactions, and possible impairment of respiratory function [53,54]. The medications administered included benzodiazepines such as lorazepam, clonazepam, and alprazolam. These drugs are metabolized in the liver by CYP450 enzymes, particularly CYP3A4. Accordingly, CYP3A4 inhibitors like Kaletra (ritonavir/lopinavir) may impair the metabolism of CYP3A4 substrates, including chlordiazepoxide, diazepam, clonazepam, flurazepam, triazolam, midazolam, and alprazolam. Some studies have reported that co-administration with ritonavir can increase the AUC of alprazolam by around 2.5-fold, resulting in heightened sedation and impairment of psychomotor performance [55,56]. In this context, the administered doses were minimal, with titration carried out in accordance with psychiatrists’ recommendations and under strict monitoring.

HD patients who initiated anxiolytic treatment de novo during COVID-19 hospitalization were those with longer hospital stays, predominantly male. This latter finding contrasts with most reports in the literature, where the requirement for anxiolytics is typically higher among female patients [57]. Paradoxically, the need for anxiolytics was higher among younger patients, particularly those with a longer history of HD. This observation aligns with the “well-being paradox,” whereby older adults exhibit higher emotional well-being than younger adults, despite a greater likelihood of health issues, physical limitations, and bereavement [58]. Our multivariable regression model identified long hospital stay and corticosteroid therapy as predictors of antidepressant use, with corticosteroid treatment—within the limitations imposed by the relatively small sample size—increasing the likelihood of requiring anxiolytic therapy by over 21-fold. We acknowledge the high odds ratio observed for corticosteroid therapy (OR: 20.99). This likely reflects the strong clinical association between corticosteroid use and in-hospital anxiolytic administration rather than model instability. Variance Inflation Factor (VIF) values for all covariates ranged from 1 to 1.3, indicating no significant multicollinearity and supporting the stability of the model estimates. The model was parsimonious, including BMI, Kt/V, corticosteroid therapy, and disease severity, chosen for clinical relevance and univariate associations. Alternative models replacing BMI and Kt/V with age and gender showed slightly poorer fit, while the association with corticosteroid therapy remained consistent. Dose–response relationships could not be evaluated because only binary corticosteroid use was recorded. Overall, these analyses support the robustness of the observed association. Although in linear regression, anxiolytic treatment was correlated with severe forms of COVID-19 and could thus serve as a potential prognostic indicator, this association was not confirmed in the multivariable analysis. Larger studies on broader population samples are required to validate these findings.

### 4.4. Mortality in the Context of COVID-19 Among HD Patients: The Role of Vaccination in the Course of SARS-CoV-2 Infection

Mortality among HD patients is significantly higher than in the general population [59]. COVID-19 infection acted as a final blow, further contributing to the exponential increase in mortality in this population [60,61]. However, in our patient cohort, over the long term, although the survival curve declined sharply for infected patients compared with COVID-negative patients during the initial period corresponding to active COVID-19 illness, no statistically significant differences were observed thereafter.

In-hospital mortality was significantly higher among patients with COVID-19 compared to non-hospitalized patients, despite the use of antiviral therapy, confirming the substantial impact of severe SARS-CoV-2 infection on the survival of HD patients. COVID-related death resulted from a combination of factors, including advanced age, higher BMI, severe systemic inflammation, respiratory compromise, acute metabolic imbalances, and the absence of vaccination. These findings are consistent with international reports documenting increased mortality among dialysis patients infected with SARS-CoV-2.

An important finding of this study is the significant reduction in COVID-19 mortality among vaccinated patients. Although the immune response to vaccination is often diminished in patients with HD, the clinical benefit remains evident, even in the presence of multiple comorbidities. In fact, COVID-19 vaccination was the only factor shown to meaningfully affect the mortality curve during the pandemic [60,62,63]. These findings strongly support current recommendations on vaccination and booster doses for vulnerable populations and underscore vaccination’s critical role as a public health intervention to reduce COVID-19-associated mortality.

The study demonstrates several notable strengths. The study’s originality lies in incorporating anxiety assessment in a retrospective pandemic study of HD patients within the broader context of evaluating their prognosis. The results of our study highlight the need for a multidisciplinary approach to managing HD patients during the pandemic, encompassing the assessment of risks associated with comorbidities, clinical severity, and psychological status, which are important components of disease prognosis. This study opens the way to a new, holistic perspective on the HD patient in critical situations, emphasizing the need to assess and address psychological alterations, particularly anxiety. Given the impact of anxiety on patient outcomes through impaired self-perception and social interaction, we underscore the necessity of developing hemodialysis-specific scales for the assessment of anxiety and depression, applicable both in daily life and in critical clinical contexts.

We acknowledge that this study has several limitations. Its retrospective, observational design restricts the comprehensiveness of the available data and precludes causal inference. Because it is a single-center, cohort-based study, the findings may not be generalizable to broader populations. The study is also subject to potential selection and survivorship biases, and the sample size, limited to patients with sufficiently complete data, was relatively small. The relatively small sample size, particularly in the hospitalized subgroup, restricted the complexity of multivariable models and increased the risk of residual confounding. To minimize overfitting, only parsimonious regression models were constructed. Although adjustments were performed for selected severity markers, unmeasured factors such as frailty, cognitive status, or psychosocial stressors may have influenced both anxiolytic use and outcomes. Furthermore, definitive data regarding anxiety diagnoses, assessment scales, or psychiatric consultations were unavailable; outcomes were inferred primarily from documented use of anxiolytic medications. In addition, due to the lack of other diagnostic instruments, anxiety was defined by the need for anxiolytic therapy during COVID-19 hospitalization. The strong association between corticosteroid therapy and anxiolytic requirement may reflect both the known neuropsychiatric effects of corticosteroids and residual confounding related to disease severity. Given the relatively small sample size, this finding should be interpreted cautiously. Post-hospitalization psychological outcomes could not be assessed. A potential limitation of our study is the influence of different pandemic waves associated with SARS-CoV-2 variants of varying severity. Although vaccination appeared to be the main driver of improved survival, we cannot entirely exclude that lower mortality among vaccinated patients was partly due to the predominance of milder variants during later waves.

## 5. Conclusions

COVID-19 in HD patients is a multidimensional pathology, in which clinical severity, psychological status, and preventive strategies, such as vaccination, significantly influence survival. Acute anxiety requiring pharmacologic treatment was highly prevalent among hospitalized HD patients with COVID-19. However, anxiolytic use was not associated with an increase in in-hospital mortality. These findings highlight the substantial psychological burden of COVID-19 in this vulnerable population and underscore the need for integrated mental health strategies in dialysis care.

## Figures and Tables

**Table 1:** The main characteristics of hospitalized versus non-hospitalized COVID-19-positive patients undergoing chronic HD.

Parameter	Hospitalized n = 80	Non-Hospitalized n = 69	*p*
Age (years)	67.638 ± 10.934	65.957 ± 12.756	0.388
Gender (male)	46 (57.5%)	34 (49.27%)	0.766
BMI	27.050 ± 6.412	26.043 ± 5.369	0.394
BSA	1.902 ± 0.262	1.853 ± 0.241	0.235
HD vintage	6.475 ± 5.625	7.662 ± 5.468	0.197
Kt/V	1.524 ± 0.323	1.616 ± 0.293	0.074
vascular access	54 (67.5%)	47 (68.11%)	0.936
DM	29 (36.25%)	21 (30.43%)	0.453
AHT	71 (88.75%)	65 (94.2%)	0.240
CAD	34 (42.5%)	29 (42.02%)	0.954
Hb (g/dL)	10.636 ± 1.616	10.713 ± 1.397	0.758
iPTH (pg/mL)	321.15 ± 326.56	387.75 ± 357.67	0.254
Ca (mg/dL)	8.88 ± 0.988	8.968 ± 0.94	0.585
P (mg/dL)	5.193 ± 2.058	5.037 ± 1.626	0.620
Hs CRP (mg/dL)	109.121 ± 99.298	23.568 ± 31.89	<0.001
Albumin (g/L)	3.68 ± 0.605	3.811 ± 0.388	0.129
T-chol (mg/dL)	153.988 ± 41.072	152.362 ± 37.762	0.803
AVT	74 (92.5%)	0	<0.001
AxT	52 (65%)	N/A	
vaccination	17 (21.25%)	63 (91.3%)	<0.001
Death	56 (70%)	33 (43.47%)	<0.006

Legend: BMI—body mass index; BSA—body surface area; HD—hemodialysis; Kt/V—dialysis efficacy; DM—diabetes mellitus; AHT—arterial hypertension; CAD—coronary artery disease; Hb—hemoglobin; iPTH—intact parathormone; ca—calcium; P—phosphorus; hs-CRP—high sensitive C reactive protein; T-chol—total cholesterol; AVT—antiviral treatment; AxT—anxiolytic treatment.

**Table 2:** Comparative characteristics of HD patients hospitalized with severe versus moderate COVID-19.

Parameter	Severe/Critical Disease n = 36	Moderate/Mild Disease n = 44	*p*
age	66.861 ± 1.882	68.273 ± 1.616	0.569
Gender (male)	22 (66.66%)	24 (54.54%)	0.555
BMI	27.886 ± 1.137	26.055 ± 0.906	0.206
HD vintage	6.028 ± 0.914	6.841 ± 0.871	0.524
vascular access	23 (63.88%)	31 (70.45%)	0.533
DM	17 (47.22%)	12 (27.27%)	0.065
AHT	31 (86.11%)	40 (90.9%)	0.449
CAD	19 (52.77%)	15 (34.09%)	0.093
malignancies	11 (30.55%)	4 (9.09%)	0.014
Hb (g/dL)	10.357 ± 0.267	10.86 ± 0.243	0.164
iPTH pg/mL	281.389 ± 46.133	353.68 ± 54.59	0.328
Ca mg/dL	8.69 ± 0.156	9.032 ± 0.153	0.130
P mg/dL	5.537 ± 0.355	4.911 ± 0.298	0.177
Hs CRP (mg/dL)	144.066 ± 16.799	80.408 ± 13.479	0.004
Albumin (g/L)	3.581 ± 0.125	3.761 ± 0.068	0.189
D dimer (ng/mL)	1908.68 ± 1788.47	1702.074 ± 1530.948	0.676
procalcitonin	8.499 ± 2.787	2.444 ± 0.669	0.031
Hospitalization days	14.75 ± 6.909	10.477 ± 6.54	0.006
AVT	31 (86.11%)	43 (97.72%)	0.050
LPV/RTV	18 (50%)	22 (50%)	1
remdesivir	7 (19.44%)	4 (9.09%)	0.181
FPV	5 (13.88%)	7 (15.9%)	0.801
DRV/RTV	2 (5.55%)	4 (9.09%)	0.550
MPV	1 (2.77%)	9 (20.45%)	0.017
Carbapenemi	24 (66.66%)	14 (31.81%)	0.002
cefalosporine	11 (30.55%)	24 (54.54%)	0.031
anticitokine	6 (16.16%)	2 (4.54%)	0.072
anxiolytic	29 (80.55%)	23 (52.27%)	0.008
anticoagulants	29 (80.55%)	35 (79.54%)	0.911
vaccination	6 (16.66%)	11 (25%)	0.365
Death	23 (63.88%)	10 (22.72%)	<0.001

Legend: BMI—body mass index; BSA—body surface area; HD—hemodialysis; Kt/V—dialysis efficacy; DM—diabetes mellitus; AHT—arterial hypertension; CAD—coronary artery disease; Hb—hemoglobin; iPTH—intact parathormone; Ca—calcium; P—phosphorus, hs-CRP—high sensitive C reactive protein; AVT—antiviral treatment; LPV/RTV—lopinavir/ritonavir; DRV/RTV—darunavir + ritonavir; MPV—molnupiravir.

**Table 3:** Comparative evaluation of mortality in hospitalized COVID-19 patients.

Parameter	Death COVID-19 n = 33	Survivors COVID-19 n = 47	*p*
age	69.364 ± 1.973	66.426 ± 1.546	0.239
gender	23 (66.69%)	23 (48.93%)	0.064
BMI	26.836 ± 1.120	26.909 ± 0.943	0.961
HD vintage	5.182 ± 4.733	7.383 ± 6.060	0.085
vascular access	22 (66.66%)	32 (68.08%)	0.894
Kt/V	1.501 ± 0.357	1.540 ± 0.299	0.596
DM	13 (39.39%)	16 (34.04%)	0.624
AHT	26 (78.78%)	45 (95.74%)	0.018
CAD	16 (48.48%)	18 (38.29%)	0.364
malignancies	8 (24.24%)	7 (14.89%)	0.292
Hb (g/dL)	10.410 ± 1.562	10.794 ± 1.651	0.299
Ca (mg/dL)	8.624 ± 1.007	9.060 ± 0.943	0.051
Hs CRP (mg/dL)	151.515 ± 116.068	79.356 ± 73.262	0.001
Albumin (g/L)	3.529 ± 0.757	3.786 ± 0.450	0.061
D dimer (ng/mL)	2306.533 ± 2044.151	1536.226 ± 1347.743	0.134
procalcitonin	10.425 ± 3.507	2.382 ± 0.591	0.005
Hospitalization days	14.061 ± 7.858	11.234 ± 6.151	0.075
AVT	28 (84.84%)	46 (97.87%)	0.029
LPV/RTV	15 (45.45)	25 (53.19%)	0.496
remdesivir	8 (24.24%)	3 (6.38%)	0.022
FPV	5 (15.15)	7 (14.89%)	0.975
DRV/RTV	1 (3.03)	5 (10.63%)	0.203
MPV	0	10 (21.26%)	0.005
Carbapenemi	19 (57.57%)	19 (40.42%)	0.130
anticitokine	4 (12.12)	4 (8.51%)	0.596
anticoagulants	26 (78.78%)	38 (80.85%)	0.820
Severe COVID-19			
vaccination	2 (6.06%)	15 (31.91%)	0.005

Legend: BMI—body mass index; BSA—body surface area; HD—hemodialysis; Kt/V—dialysis efficacy; DM—diabetes mellitus; AHT—arterial hypertension; CAD—coronary artery disease; Hb—hemoglobin; iPTH—intact parathormone; Ca—calcium; P—phosphorus, hs-CRP—high sensitive C reactive protein; AVT—antiviral treatment; LPV/RTV—lopinavir/ritonavir; DRV/RTV—darunavir + ritonavir; MPV—molnupiravir.

**Table 4:** Predictors for COVID-19 mortality in hospitalized patients.

Predictor	OR	95%CI	*p*
Age	1.042	0.990–1.096	0.113
hs CRP	1.008	1.002–1.014	0.008
Antiviral treatment	0.058	0.004–0.813	0.035
vaccination	0.179	0.032–0.899	0.037

**Table 5:** Predictors for COVID-19 mortality in hospitalized patients using GLM module for multivariable regression.

Predictor	OR	95%CI	*p*
Age	1.008	0.999–1.017	0.088
hs CRP	1.002	0.001–0.003	0.002
Antiviral treatment	0.607	0.421–0.877	0.009
vaccination	0.749	0.589–0.951	0.037

**Table 6:** De novo anxiolytic use during hospitalization.

Parameters	“De Novo” Anxiolytic Treatment n = 52	No Anxiolytics n = 28	*p*
Hospitalization days	14.712 ± 7.047	8.107 ± 4.491	<0.001
age	66.712 ± 11.185	69.357 ± 10.429	0.305
gender	31 (67.39%)	15 (53.57%)	0.602
HD vintage	6.981 ± 5.561	5.536 ± 5.725	0.276
BMI	27.875 ± 6.320	25.029 ± 6.276	0.058
Hb	10.518 ± 1.766	10.854 ± 1.293	0.379
albumin	3.669 ± 0.650	3.700 ± 0.523	0.832
Kt/V	1.484 ± 0.307	1.598 ± 0.344	0.133
hs-CRP	109.605 ± 95.08	108.224 ± 108.486	0.953
AVT	49 (66.21%)	25 (33.78)	0.423
anticoagulants	43 (67.18%)	21 (32.81%)	0.412
corticotherapy	48 (82.75%)	10 (17.24%)	<0.001
COVID-19 severe form	29 (80.55%)	7 (19.44%)	0.008
ICU admission	18 (81.81%)	4 (18.18%)	0.052
COVID-19 death	21 (40.38%)	12 (42.85%)	0.903

Legend: BMI—body mass index; HD—hemodialysis; Kt/V—dialysis efficacy; Hb—hemoglobin; hs-CRP—high sensitive C reactive protein; AVT—antiviral treatment; ICU—intensive care unit.

**Table 7:** Model coefficients for anxiolytics correlations during COVID-19 hospitalization.

Predictor	OR	95%CI	*p*
gender	0.054	0–7.387	0.245
BMI	1.051	0.950–1.163	0.478
Hb	0.993	0.682–1.447	0.971
severe form COVID-19	1.906	0.548–6.624	0.310
corticotherapy	16.403	4.332–62.111	<0.001

**Table 8:** GLM module for multivariable regression; model coefficients for anxiolytics correlations during COVID-19 hospitalization.

Predictor	OR	95%CI	*p*
gender	1.030	0.864–1.23	0.733
BMI	1.007	0.993–1.021	0.357
Hb	0.999	0.946–1.055	0.974
severe form COVID-19	1.096	0.909–1.322	0.329
corticotherapy	1.805	1.464–2.228	<0.001

**Table 9:** Contingency table anxiolytics/corticosteroids.

	Anxiolytics (yes)	Anxiolytics (no)	Total
Corticosteroids (yes)	48	10	58
Corticosteroids (no)	4	18	22
total	52	28	80

**Table 10:** Comparative Characteristics of COVID-Positive and COVID-Negative Patients in a Hemodialysis Program.

Parameter	COVID+ n = 149	COVID- n = 62	*p*-Value
age	66.859 ± 11.802	62.774 ± 2.044	0.042
Gender (male)	84 (56.37%)	34 (54.83%)	0.838
BMI	26.492 ± 5.947	24.156 ± 6.501	0.012
BSA	1.870 ± 0.253	1.782 ± 0.278	0.015
HD vintage	7.020 ± 5.567	6.694 ± 5.312	0.695
Kt/V	1.567 ± 0.312	1.591 ± 0.323	0.622
vascular access	101 (67.78%)	38 (61.29%)	0.365
DM	50 (33.55%)	18 (29.03%)	0.522
AHT	136 (91.27%)	56 (90.32%)	0.826
CAD	63 (42.28%)	23 (37.09%)	0.485
malignances	25 (16.77%)	9 (14.51%)	0.684
Hb (g/dL)	10.671 ± 1.514	10.778 ± 1.472	0.641
iPTH pg/mL	329.693 ± 320.6	432.2 ± 577.151	0.212
Ca (mg/dL)	8.920 ± 0.964	8.880 ± 0.922	0.782
P (mg/dL)	5.123 ± 1.872	4.995 ± 1.804	0.653
Albumin (g/L)	3.740 ± 0.520	3.723 ± 0.371	0.817
T chol (mg/dL)	153.235 ± 39.450	151.233 ± 147.5	0.740
vaccination	52 (34.89%)	29 (46.77%)	0.106
Death	89 (59.73%)	32 (51.61%)	0.277

Legend: BMI—body mass index; BSA—body surface area; HD—hemodialysis; Kt/V—dialysis efficacy; DM—diabetes mellitus; AHT—arterial hypertension; CAD—coronary artery disease; Hb—hemoglobin; iPTH—intact parathormone; Ca—calcium; P—phosphorus; T-chol-total cholesterol.

**Table 11:** Cox multivariable analysis for HD patients.

Parameters	*p*	HR	95%CI
age	0.246	1.012	0.992–1.032
BMI	0.895	1.003	0.962–1.046
COVID (+)	0.96	1.27	0.988–2.394
HD vintage	0.154	0.970	0.930–1.012
kT/V	0.345	1.398	0.698–2.800
hemoglobin	0.201	0.812	0.758–1.060
albumin	0.359	0.812	0.520–1.267
Severe form COVID-19	0.011	2.086	1.180–3.686
Antiviral treatment	0.346	0.780	0.465–1.308
SARS-CoV2 vaccination	0.009	0.472	0.269–0.826

## Data Availability

The data presented in this study are available on request from the corresponding author. The data are not publicly available due to ethical reasons.

## Supplementary Materials

The following supporting information can be downloaded at: https://www.mdpi.com/article/10.3390/medicina62040744/s1, Table S1: Antiviral pharmacotherapy in HD patients hospitalized with COVID-19.

## References

[1] Ratiu I.A., Babes V.V., Hocopan O., Ratiu C.A., Croitoru C.A., Moisa C., Blaj-Tunduc I.P., Marian A.M., Babeș E.E. (2025). The Burden of Heart Failure in End-Stage Renal Disease: Insights from a Retrospective Cohort of Hemodialysis Patients. J. Clin. Med..

[2] Kaur R., Singh R. (2022). Mechanistic insights into CKD-MBD-related vascular calcification and its clinical implications. Life Sci..

[3] Covic A., Vervloet M., Massy Z.A., Torres P.U., Goldsmith D., Brandenburg V., Mazzaferro S., Evenepoel P., Bover J., Apetrii M. (2018). Bone and mineral disorders in chronic kidney disease: Implications for cardiovascular health and ageing in the general population. Lancet Diabetes Endocrinol..

[4] Kakani E., Elyamny M., Ayach T., El-Husseini A. (2019). Pathogenesis and management of vascular calcification in CKD and dialysis patients. Semin. Dial..

[5] Ratiu I.A., Babeș E.E., Georgescu L.M., Hocopan O., Dejeu D., Moisa C., Gavra D.N., Ratiu C.A. (2026). Understanding the Drivers of Hypothyroidism in Patients Undergoing Chronic Hemodialysis. Diagnostics.

[6] Wu H.C., Tseng S.F., Wang W.J., Chen H.J., Lee L.C. (2017). Association between obesity with low muscle mass and dialysis mortality. Intern. Med. J..

[7] Ratiu I.A., Moisa C.F., Țiburcă L., Hagi-Islai E., Ratiu A., Bako G.C., Ratiu C.A., Stefan L. (2024). Antimicrobial Treatment Challenges in the Management of Infective Spondylodiscitis Associated with Hemodialysis: A Comprehensive Review of Literature and Case Series Analysis. Antibiotics.

[8] Marc L., Mihaescu A., Lupusoru R., Schiller O., Bob F., Chisavu L., Bende F., Sirli R., Schiller A. (2023). Hepatitis C and hepatitis B virus infection in hemodialysis patients after nationwide direct antiviral agents therapy-experience of 10 Romanian HD centers. Int. Urol. Nephrol..

[9] Graterol Torres F., Molina M., Soler-Majoral J., Romero-González G., Rodríguez Chitiva N., Troya-Saborido M., Socias Rullan G., Burgos E., Paúl Martínez J., Urrutia Jou M. (2022). Evolving Concepts on Inflammatory Biomarkers and Malnutrition in Chronic Kidney Disease. Nutrients.

[10] Ebert T., Neytchev O., Witasp A., Kublickiene K., Stenvinkel P., Shiels P.G. (2021). Inflammation and Oxidative Stress in Chronic Kidney Disease and Dialysis Patients. Antioxid. Redox Signal..

[11] Campo S., Lacquaniti A., Trombetta D., Smeriglio A., Monardo P. (2022). Immune System Dysfunction and Inflammation in Hemodialysis Patients: Two Sides of the Same Coin. J. Clin. Med..

[12] Antonescu A., Vicaș S., Teușdea A.C., Rațiu I.A., Antonescu I.A., Micle O., Vicaș L., Mureșan M., Gligor F. (2014). The levels of serum biomarkers of inflammation in hemodialysis patient. Farmacia.

[13] Ng H.J., Tan W.J., Mooppil N., Newman S., Griva K. (2015). Prevalence and patterns of depression and anxiety in hemodialysis patients: A 12-month prospective study on incident and prevalent populations. Br. J. Health Psychol..

[14] World Health Organization Depression. https://www.who.int/news-room/fact-sheets/detail/depression.

[15] Gerogianni G., Lianos E., Kouzoupis A., Polikandrioti M., Grapsa E. (2018). The role of socio-demographic factors in depression and anxiety of patients on hemodialysis: An observational cross-sectional study. Int. Urol. Nephrol..

[16] Schouten R.W., Haverkamp G.L., Loosman W.L., Chandie Shaw P.K., van Ittersum F.J., Smets Y.F.C., Vleming L.J., Dekker F.W., Honig A., Siegert C.E.H. (2019). Anxiety Symptoms, Mortality, and Hospitalization in Patients Receiving Maintenance Dialysis: A Cohort Study. Am. J. Kidney Dis..

[17] Du Z., Chu T., Dai F., Mao H., Zhao J. (2025). Correlation analysis of anxiety, depression, and sleep quality in end-stage renal disease patients undergoing maintenance hemodialysis. Medicine.

[18] Ma S.J., Wang W.J., Tang M., Chen H., Ding F. (2021). Mental health status and quality of life in patients with end-stage renal disease undergoing maintenance hemodialysis. Ann. Palliat. Med..

[19] Ok E., Aydin Sayilan A., Sayilan S., Sousa C.N., Ozen N. (2022). Noise levels in the dialysis unit and its relationship with sleep quality and anxiety in patients receiving HD: A pilot study. Ther. Apher. Dial..

[20] Natale P., Palmer S.C., Ruospo M., Saglimbene V.M., Rabindranath K.S., Strippoli G.F. (2019). Psychosocial interventions for preventing and treating depression in dialysis patients. Cochrane Database Syst. Rev..

[21] Friedli K., Almond M., Day C., Chilcot J., da Silva Gane M., Davenport A., Guirguis A., Fineberg N., Spencer B., Wellsted D. (2015). A study of sertraline in dialysis (ASSertID): A protocol for a pilot randomised controlled trial of drug treatment for depression in patients undergoing haemodialysis. BMC Nephrol..

[22] ClinicalTrials.gov Study NCT02238977. NCT02238977.

[23] Hedayati S.S., Yalamanchili V., Finkelstein F.O. (2012). A practical approach to the treatment of depression in patients with chronic kidney disease and end-stage renal disease. Kidney Int..

[24] Reinhold J.A., Rickels K. (2015). Pharmacological treatment for generalized anxiety disorder in adults: An update. Expert Opin. Pharmacother..

[25] Constantino J.L., Fonseca V.A. (2019). Pharmacokinetics of antidepressants in patients undergoing hemodialysis: A narrative literature review. Braz. J. Psychiatry.

[26] Nagler E.V., Webster A.C., Vanholder R., Zoccali C. (2012). Antidepressants for depression in stage 3–5 chronic kidney disease: A systematic review of pharmacokinetics, efficacy and safety with recommendations by European Renal Best Practice (ERBP). Nephrol. Dial. Transplant..

[27] Collomb M., Sens F., Sanchez S., Jolivot A., Pivot C., Juillard L., Paillet C. (2015). Long-term benzodiazepine use among dialysis patients: A descriptive study. Nephrol. Ther..

[28] Winkelmayer W.C., Mehta J., Wang P.S. (2007). Benzodiazepine use and mortality of incident dialysis patients in the United States. Kidney Int..

[29] Muzaale A.D., Daubresse M., Bae S., Chu N.M., Lentine K.L., Segev D.L., McAdams-DeMarco M. (2020). Benzodiazepines, Codispensed Opioids, and Mortality among Patients Initiating Long-Term In-Center Hemodialysis. Clin. J. Am. Soc. Nephrol..

[30] Bonenkamp A.A., Druiventak T.A., van Eck van der Sluijs A., van Ittersum F.J., van Jaarsveld B.C., Abrahams A.C. (2021). DOMESTICO Study Group. The Impact of COVID-19 on the mental health of dialysis patients. J. Nephrol..

[31] Wang Y., Kala M.P., Jafar T.H. (2020). Factors associated with psychological distress during the coronavirus disease 2019 (COVID-19) pandemic on the predominantly general population: A systematic review and meta-analysis. PLoS ONE.

[32] Yu J.Y., Kim J.S., Hong C.M., Lee K.Y., Cho N.J., Park S., Gil H.W., Lee E.Y. (2021). Psychological distress of patients with end-stage kidney disease undergoing dialysis during the 2019 coronavirus disease pandemic: A cross-sectional study in a University Hospital. PLoS ONE.

[33] Infectious Diseases Society of America COVID-19 Guideline Treatment and Management. https://www.idsociety.org/practice-guideline/covid-19-guideline-treatment-and-management/.

[34] Fisher M., Yunes M., Mokrzycki M.H., Golestaneh L., Alahiri E., Coco M. (2020). Chronic Hemodialysis Patients Hospitalized with COVID-19: Short-term Outcomes in the Bronx, New York. Kidney360.

[35] Sevinc C., Demirci R., Timur O. (2021). Predicting hospital mortality in COVID-19 hemodialysis patients with developed scores. Semin. Dial..

[36] Munoz Mendoza J., Aponte-Becerra L., Torres C., Rozas J., Francois P., Salcedo J., Barreto A., Rodriguez J., Suarez M., Fornoni A. (2023). One-year all-cause mortality in hospitalized patients with COVID-19 and end-stage renal disease undergoing hemodialysis. Clin. Nephrol..

[37] Grujic N., Pešić S., Naumovic R. (2021). MO851: Incidence and mortality of coronavirus disease (COVID-19) in hemodialysis patients. Nephrol. Dial. Transplant..

[38] Micle O., Vicas I.S., Ratiu A.I., Muresan M. (2015). Cystatin C, a low molecular weight protein in chronic renal failure. Farmacia.

[39] Rațiu I.A., Rațiu C.A., Miclăuş V., Boşca A.B., Turan Kazancioğlu R., Constantin A.M., Bako G.C., Şovrea A.S. (2021). The pioneer use of a modified PRGF-Endoret^®^ technique for wound healing in a hemodialyzed diabetic patient in a terminal stage of renal disease. Rom. J. Morphol. Embryol..

[40] Ratiu I.A., Mihaescu A., Olariu N., Ratiu C.A., Cristian B.G., Ratiu A., Indries M., Fratila S., Dejeu D., Teusdea A. (2024). Hepatitis C Virus Infection in Hemodialysis Patients in the Era of Direct-Acting Antiviral Treatment: Observational Study and Narrative Review. Medicina.

[41] RECOVERY Trial. https://www.recoverytrial.net.

[42] Angus D.C., Berry S., Lewis R.J., Al-Beidh F., Arabi Y., van Bentum-Puijk W., Bhimani Z., Bonten M., Broglio K., Brunkhorst F. (2020). The REMAP-CAP (Randomized Embedded Multifactorial Adaptive Platform for Community-acquired Pneumonia) Study. Rationale and Design. Ann. Am. Thorac. Soc..

[43] Elmekaty E., Maklad A., Abouelhassan R., Munir W., Ibrahim M.I.M., Nair A., Alibrahim R., Iqbal F., Al Bishawi A., Abdelmajid A. (2022). Efficacy of Anakinra in the Management of Patients with COVID-19 Infection: A Randomized Clinical Trial. medRxiv.

[44] Kharazmi A.B., Moradi O., Haghighi M., Kouchek M., Manafi-Rasi A., Raoufi M., Shoaei S.D., Hadavand F., Nabavi M., Miri M.M. (2022). A randomized controlled clinical trial on efficacy and safety of anakinra in patients with severe COVID-19. Immun. Inflamm. Dis..

[45] Ozkan Kurtgoz P., Sackan F., Kızılarslanoglu M.C., Bilgin O., Guney I. (2022). Effect of anxiety on COVID-19 infection in hemodialysis patients. Ther. Apher. Dial..

[46] Kahl K.G., Correll C.U. (2020). Management of Patients with Severe Mental Illness During the Coronavirus Disease 2019 Pandemic. JAMA Psychiatry.

[47] Dashiell-Earp C.N., Bell D.S., Ang A.O., Uslan D.Z. (2014). Do physicians spend less time with patients in contact isolation?: A time-motion study of internal medicine interns. JAMA Intern. Med..

[48] Purssell E., Gould D., Chudleigh J. (2020). Impact of isolation on hospitalised patients who are infectious: Systematic review with meta-analysis. BMJ Open.

[49] Korte S.M. (2001). Corticosteroids in relation to fear, anxiety and psychopathology. Neurosci. Biobehav. Rev..

[50] Nasereddin L., Alnajjar O., Bashar H., Abuarab S.F., Al-Adwan R., Chellappan D.K., Barakat M. (2024). Corticosteroid-Induced Psychiatric Disorders: Mechanisms, Outcomes, and Clinical Implications. Diseases.

[51] Liu Y., Ho R.C., Mak A. (2012). Interleukin (IL)-6, tumour necrosis factor alpha (TNF-α) and soluble interleukin-2 receptors (sIL-2R) are elevated in patients with major depressive disorder: A meta-analysis and meta-regression. J. Affect. Disord..

[52] Mak I.W., Chu C.M., Pan P.C., Yiu M.G., Ho S.C., Chan V.L. (2010). Risk factors for chronic post-traumatic stress disorder (PTSD) in SARS survivors. Gen. Hosp. Psychiatry.

[53] Coin A., Malara A., Noale M., Trevisan C., Devita M., Abbatecola A.M., Gareri P., Del Signore S., Bellelli G., Fumagalli S. (2024). Real-World Use of Trazodone in Older Persons in Long Term Care Setting: A Retrospective Study. Int. J. Geriatr. Psychiatry.

[54] Mohebbi N., Talebi A., Moghadamnia M., Nazari Taloki Z., Shakiba A. (2020). Drug Interactions of Psychiatric and COVID-19 Medications. Basic Clin. Neurosci..

[55] Hesse L.M., von Moltke L.L., Greenblatt D.J. (2003). Clinically important drug interactions with zopiclone, zolpidem and zaleplon. CNS Drugs.

[56] Dybul M., Fauci A.S., Bartlett J.G., Kaplan J.E., Pau A.K. (2002). Guidelines for using antiretroviral agents among HIV-infected adults and adolescents. Ann. Intern. Med..

[57] Kurvits K., Toompere K., Jaanson P., Uusküla A. (2024). The COVID-19 pandemic and the use of benzodiazepines and benzodiazepine-related drugs in Estonia: An interrupted time-series analysis. Child Adolesc. Psychiatry Ment. Health.

[58] Baltes P.B., Baltes M.M. (1990). Successful Aging: Perspectives from the Behavioral Sciences.

[59] Bossola M., Mariani I., Antocicco M., Pepe G., Di Stasio E. (2025). Frailty, All-Cause Mortality, and Hospitalization in Patients on Maintenance Hemodialysis: A Systematic Review and Meta-Analysis. J. Clin. Med..

[60] Ratiu I.A., Filip L., Moisa C., Ratiu C.A., Olariu N., Grosu I.D., Bako G.C., Ratiu A., Indries M., Fratila S. (2025). The Impact of COVID-19 on Long-Term Mortality in Maintenance Hemodialysis: 5 Years Retrospective Cohort Study. J. Clin. Med..

[61] Yamada T., Sakai Y., Kashiwagi T., Iwabu M. (2024). Prognostic Factors for Mortality in Maintenance Hemodialysis Patients Infected with SARS-CoV-2. J. Nippon Med. Sch..

[62] Brkovic V., Nikolic G., Baralic M., Kravljaca M., Milinkovic M., Pavlovic J., Lausevic M., Radovic M. (2024). A Study on Mortality Predictors in Hemodialysis Patients Infected with COVID-19: Impact of Vaccination Status. Vaccines.

[63] Hu R., Yin J., He T., Zhu Y., Li Y., Gao J., Ye X., Hu L., Li Y. (2024). Impact of COVID-19 Vaccination on Mortality and Clinical Outcomes in Hemodialysis Patients. Vaccines.

