# Supplementary material for: Clinical and Psychological Impact of COVID-19 on Maintenance Hemodialysis Patients: Hospitalization Burden, De Novo Anxiolytic Use, and Long-Term Survival
Source: Medicina (Kaunas). 2026 Apr 13;62(4):744. doi: 10.3390/medicina62040744 (PMC13117431; doi:10.3390/medicina62040744)
Supplement: Supplementary file 1 [file medicina-62-00744-s001.zip › medicina-4215731-supplementary.pdf]

Antiviral pharmacotherapy in HD patients hospitalized with COVID-19

The antiviral agents administered during hospitalization, depending on their availability at the time of admission, included lopinavir/ritonavir (LPV/RTV) (Kaletra), favipiravir (FPV) (FluGuard), remdesivir (RDV) (Veklury), darunavir+ritonavir (Prezista+Norvir)(DRV/RTV), and molnupiravir (MPV) (Lagevrio). The evaluation of patients who received each specific antiviral agent compared with all other patients did not reveal significant differences in age, BMI or length of hospitalization (Table S1).

Table S1. Antiviral pharmacotherapy in HD patients hospitalized with COVID-19.

**AVT**	**LPV/RTV n=40**	**FPV n=12**	**RDV n=11**	**DRV/RTV n=6**	**MPV n=10**
age	69.175±13.911<br>p=0.211	69.50±8.0<br>p=0.526	64.091±15.12<br>p=0.249	65±13.282<br>p=0.542	61.50±13.7<br>p=0.203
Hd vintage	7.02±5.318<br>Me=5.5<br>p=0.385	4.33±1.61<br>Me=4<br>p=0.154	6.364±6.33<br>Me=4<br>p=0.944	5±3.52<br>Me=5<br>p=0.508	9±8.537<br>Me=6.5<br>p=0.130
BMI	27.43±6.98<br>p=0.445	27.85±5.49<br>p=0.543	24.6±7.04<br>p=0.245	28.08±6.15<br>p=0.635	26.8±7.13<br>p=0.967
Severe disease	p=1	p=0.8	p=0.181	P=0.55	OR=0.11<br>95%CI<br>[0.013-0.92]<br>*P=0.017*
Hb (g/dl)	10.84±1.63<br>p=0.249	10.41±1.18<br>p=0.601	9.58±1.69<br>p=*0.018*	10.647±2<br>p=0.792	11.77±1.03<br>p=*0.017*
hs CRP (mg/dl)	105.45±96.83<br>p=0.743	89.29±69.12<br>p=0.457	222.81±115.7<br>p*<0.001*	54.33±41.38<br>p=0.161	40.53±41.17<br>p=*0.019*
Albumin (g/l)	3.75±0.46<br>p=0.271	3.69±0.50<br>p=0.934	3.05±0.87<br>p*<0.001*	3.835±0.52<br>p=0.517	3.889±0.243<br>p=0.245
D dimer (ng/ml)	1652.5±1441.8<br>p=0.627	1168.9±1128.5<br>p=0.177	2117.7±2102.9<br>p=0.565	2523±2899<br>p=0.423	1709±1628<br>p=0.892
procalcitonin	3.43±5.09<br>p=0.164	2.1±1.51<br>p=0.478	12.28±10.68<br>p=0.058	12.06±26.77<br>p=0.094	1.224±1.38<br>p=0.204
Hospitalization days	13.4±7.31<br>Me=12.5<br>p=0.203	9.92±4.6<br>Me=9<br>p=0.184	16.36±6.07<br>Me=17<br>p=*0.042*	14.17±7.55<br>Me=12.5<br>p=0.524	7.4±4.27<br>*Me=7*<br>p=*0.015*
Evolution	p=0.496	p=0.975	OR=4.693<br>95%CI [1.14-19.316]<br>p=0.022	0.203	OR=0.053[a]<br>95%CI<br>[0.003-0.945]<br>p=0.005

[a] Haldane-Anscombe correction

Legend: AVT- antiviral tratment; BMI- body mass index; HD- hemodialysis; Hb- hemoglobin; hs-CRP- high sensitive C reactive protein; LPV/RTV- lopinavir/ritonavir; DRV/RTV- darunavir+ritonavir; MPV- molnupiravir.
